# Web-based machine learning application for interpretable prediction of prolonged length of stay after lumbar spinal stenosis surgery: a retrospective cohort study with explainable AI

**DOI:** 10.3389/fphys.2025.1542240

**Published:** 2025-02-19

**Authors:** Paierhati Yasheng, Alimujiang Yusufu, Yasenjiang Yimiti, Haopeng Luan, Cong Peng, Xinghua Song

**Affiliations:** ^1^ Department of Spine Surgery, The Sixth Affiliated Hospital of Xinjiang Medical University, Urumqi, Xinjiang, China; ^2^ Department of Spine Surgery, The First Affiliated Hospital of Xinjiang Medical University, Urumqi, Xinjiang, China; ^3^ The First People’s Hospital of Kashi Prefecture, Kashi, Xinjiang, China

**Keywords:** lumbar spinal stenosis, postoperative length of stay, spine surgery, interpretable model, SHAP value

## Abstract

**Objectives:**

Lumbar spinal stenosis (LSS) is an increasingly important issue related to back pain in elderly patients, resulting in significant socioeconomic burdens. Postoperative complications and socioeconomic effects are evaluated using the clinical parameter of hospital length of stay (LOS). This study aimed to develop a machine learning-based tool that can calculate the risk of prolonged length of stay (PLOS) after surgery and interpret the results.

**Methods:**

Patients were registered from the spine surgery department in our hospital. Hospital stays greater than or equal to the 75th percentile for LOS was considered extended PLOS after spine surgery. We screened the variables using the least absolute shrinkage and selection operator (LASSO) and permutation importance value and selected nine features. We then performed hyperparameter selection via grid search with nested cross-validation. Receiver operating characteristics curve, calibration curve and decision curve analysis was carried out to assess model performance. The result of the final selected model was interpreted using Shapley Additive exPlanations (SHAP), and Local Interpretable Model-agnostic Explanations (LIME) were used for model interpretation. To facilitate model utilization, a web application was deployed.

**Results:**

A total of 540 patients were involved, and several features were finally selected. The final optimal random forest (RF) model achieved an area under the curve (ROC) of 0.93 on the training set and 0.83 on the test set. Based on both SHAP and LIME analyses, intraoperative blood loss emerged as the most significant contributor to the outcome.

**Conclusion:**

Machine learning in association with SHAP and LIME can provide a clear explanation of personalized risk prediction, and spine surgeons can gain a perceptual grasp of the impact of important model components. Utilization and future clinical research of our RF model are made simple and accessible through the web application.

## 1 Introduction

Lumbar spinal stenosis (LSS) is one of the common spinal diseases, which is a well-known cause of back pain, leg pain, and neurogenic intermittent claudication (Katz et al., 2022). It is estimated that 103 million people are suffering from LSS worldwide, bringing a great socioeconomic burden on families (Won et al., 2022; Ravindra et al., 2018). The treatment of LSS can be categorized into conservative, interspinous spacers, epidural steroid injections, and surgical decompression with or without fusion (Paisley et al., 2012; Phan et al., 2016). However, surgical decompression is still regarded as the most effective option (Davis et al., 2017). Surgery for LSS requires more medical costs and, as a result, costs more money than nonoperative options (Cairns et al., 2019). One of the causes of the cost rise is the duration of the postoperative length of stay in the hospital, which is also an indirect indication of surgical recovery and postoperative problems. In other words, a prolonged length of stay (PLOS) after surgery suggests a delayed post-spine surgery recovery.

Previous research regarding the prolonged length of stay has been reported about fusion and laminectomy (decompression) surgeries (Lu et al., 2022; Saravi et al., 2022; Basques et al., 2014). Independent risk factors, including age, American Society of Anesthesiologists class (ASA), preoperative hematocrit, body mass index, number of affected levels, liposomal bupivacaine, operation time, etc., have been explained (Roh et al., 2020). Additionally, it was thought that the enhanced recovery after surgery (ERAS) protocol was a preferable option as it has been demonstrated to speed up the recovery of physiological function and decrease early postoperative discomfort, problems, and hospital length of stay (LOS) (Dietz et al., 2019; Porche et al., 2022). Even though some of the abovementioned research used complex approaches, there are still some limitations when it comes to clinical practice as the lack of utilized explanation approach.

The term machine learning describes a group of computer science-based techniques that employ data patterns to recognize or forecast outcomes. Machine learning (ML) techniques for predictive modeling have recently attracted more attention. While some ML algorithms have been around for a while, their use for predicting new data from patterns that have already been identified has only recently attracted significant attention. This has allowed researchers to identify patterns that are difficult to recognize from complex combinations of multiple biomarkers. The rise in ML usage can be attributed to the emergence of the big data era as well as to the creation of new algorithms and gradually increasing processing capacity. It offers a potent set of tools to define and may automatically create associations between the traits and outcomes of interest, especially when they are nonlinear and complex, by analyzing the available data and maximizing performance criteria. Complex non-linear machine learning models, on the other hand, have a reputation for being a black box (inadequate interpretation) that fails to reveal the elements influencing a prediction in situations when the majority of clinical patterns identified in data are non-linear (Leidner et al., 2019; Marko et al., 2017). Besides, insufficient model applicability in clinical practice is also the main issue that needs to be solved (Zhang et al., 2018; Jain and Potdar, 2021).

It is reported that medical data is increasing up to 48% annually, and surges in data pose challenges for its proper utilization in improving patient care, thus leading to the creation of numerous new tools that rely on artificial intelligence (AI) and machine learning (ML) (Gunzer et al., 2022). The rapid advancement in computing power and accessibility has triggered a technological revolution in medicine that is already altering various aspects of the field, through the integration of AI and ML. ML is commonly employed in various health-related tasks, such as the integration of multiple variables to emulate human clinical decision-making skills, automation of testing and treatment algorithms, recognition and interpretation of patterns from imaging data, and monitoring trends in test utilization. There is a noticeable necessity to implement systematic principles of data science that are rationally driven to manage the constantly expanding collection of qualitative and quantitative elements of medical information and classification.

To address these shortcomings, this study combined the advanced ML algorithm that includes more relevant features available during the perioperative management period with SHapley Additive exPlanations (SHAP) and other techniques for model interpretation (Linardatos et al., 2020; Lundberg et al., 2020). What is more, to improve the applicability of our final selected optimal model, we deployed our model on an online website.

## 2 Methods

The study cohort included patients who had undergone spine surgery at our hospital. Records were de-identified for this study, and informed consent was not required for this retrospective study, which was approved (K202309-15) by the institutional ethics committee board of Xinjiang Medical University Affiliated First Hospital.

### 2.1 Study population

This was a retrospective study in which we enrolled patients who underwent open decompression and fusion surgery between January 2019 and November 2022, meeting the inclusion and exclusion criteria. Decompression surgery is indicated for patients exhibiting spinal stenosis refractory to conservative interventions, provided that comprehensive clinical and radiological assessment has detected no evidence of spinal instability (Fritsch et al., 2017). Fusion as a treatment modality for primary disc herniation is generally rare, except in situations where recurrent herniation remains unresponsive to decompressive or discectomy procedures, or in circumstances where other factors such as spinal stenosis with instability, spinal deformity, or adjacent segment disease occurring from prior spinal fusion operations are concomitant with concurrent disc herniation at the identical level (Wu PH. et al., 2020). We enrolled patients who underwent both decompression and fusion because they accounted for the vast majority of patients.

The inclusion criteria were as follows: (Katz et al., 2022): aged ≥18 years (Won et al., 2022); symptom of neurogenic claudication (Ravindra et al., 2018); underwent open surgery (decompression and fusion); and (Paisley et al., 2012) had radiological (magnetic resonance imaging, MRI) assessment. Most of the enrolled patients who had been admitted to our department had severe symptoms of nerve compression and vertebra instability.

The exclusion criteria were as follows: (Katz et al., 2022): age less than 18 years (Won et al., 2022); complications with malignant tumors (Ravindra et al., 2018); complications with lumbar spondylolisthesis or lumbar spine fractures (Paisley et al., 2012); complications with spine infectious diseases (Phan et al., 2016); with spine deformity (Davis et al., 2017); with thrombosis (Cairns et al., 2019); patients transferred to the intensive care unit (ICU).

### 2.2 Data collection

**FIGURE 1:**
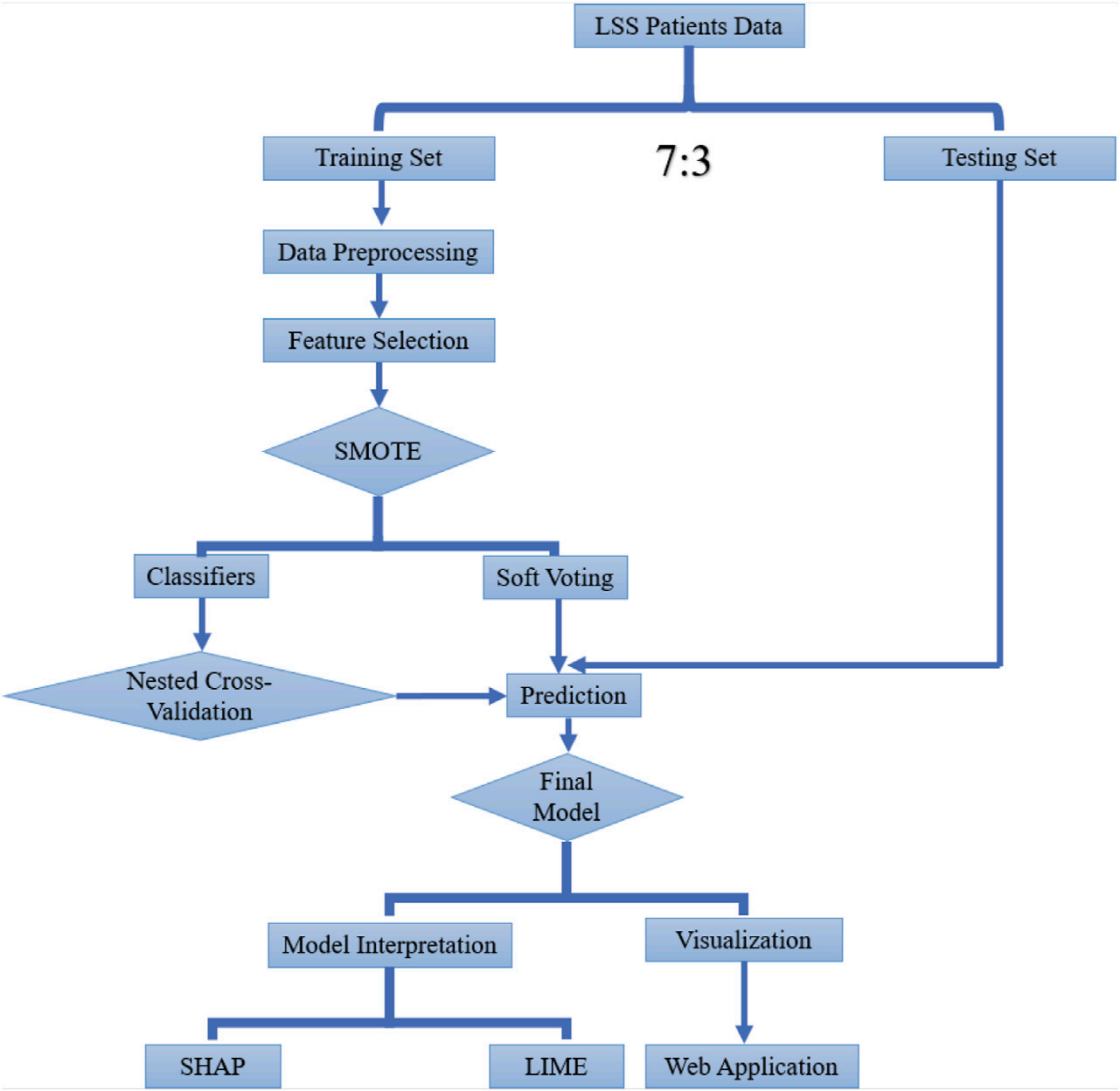
Workflow of this study.

To investigate the risk of PLOS after surgery, we extracted all available factors related to PLOS in the perioperative management period, including basic information (age, gender, ethnicity, body mass index [BMI], smoking, and alcohol), preoperative stage factors (symptom durations, affected limb, muscle strength, erythrocyte sedimentation rate [ESR], C-reactive protein [CRP], white blood cell [WBC], hemoglobin, preoperative albumin [ALb], gamma-glutamyl transferase [GGT], alanine aminotransferase [ALT], aspartate aminotransferase [AST], and alkaline phosphatase [ALP]), and preexisting conditions (hypertension, diabetes mellitus [DM], cerebrovascular, cardiovascular, hepatic, kidney, thyroid, and respiratory diseases). Information related to the procedure was also included, such as the extent of involvement, how the surgery went on that day, how many vertebrae were affected, how long the surgery took, how much fluid was administered, the amount of blood transfused, and the amount of blood lost. Postoperative details, including the amount of drainage on day one, were also recorded. 70% of the study participants were assigned at random to the training set, while the remaining participants were split into the testing set (Figure 1).

### 2.3 PLOS after surgery

The number of days a patient stays in the hospital following spine surgery, from the day of the procedure until they are released, is referred to as the hospital length of stay. The target classes were classified as prolonged when the hospital length of stay was higher than the 75% percentile and normal when lower than the 75% percentile (binary classification task).

### 2.4 Dimension reduction and data preprocessing

Once the feature vectors from each data source have been combined, we can either use them directly as input to the classification algorithms or use a feature selection algorithm to remove redundant or correlated features while keeping a more useful subset. This also helps with classification tasks, visualization, and web application development. We choose to utilize univariate analysis and the least absolute shrinkage and selection operator (LASSO) to choose variables to reduce the dimension of features. LASSO will punish some unimportant feature coefficients to zero (Vasquez et al., 2016). Besides, permutation feature importance calculated by random forest (RF) was also taken into consideration when screening the features (Orlenko and Moore, 2021). Permutation feature importance overcomes the limitations of impurity-based feature importance (Gini importance), which has a bias toward high-cardinality features and can be computed on a left-out test set. Furthermore, the impurity-based feature importance for trees is strongly biased and favors high cardinality features (typically numerical features) over low cardinality features such as binary features or categorical variables with a small number of possible categories. Missing data value was imputed via random-forest-based imputation (Hong and Lynn, 2020).

Since there is an imbalance in the dataset when comparing the ratio of positive to negative classes, we further investigate data rebalancing. According to earlier research on the effects of class imbalance, classifier biases could result from an imbalanced dataset, which would subsequently increase the rate of misclassification and degrade the classification model. Based on this, we devised a synthetic minority oversampling method (SMOTE) to balance the data by oversampling the minority class (Nakamura et al., 2013; Exarchos et al., 2012).

### 2.5 Model development

In this research, eight models were implemented in total. Supervised machine learning (ML) algorithms utilize training data to create a function (f) that maps input variables/features (X) to output/target (Y), with the use of “labeled” training data sets is a common feature of supervised ML platforms in providing either a qualitative or quantitative output. The labeled nature of these data sets during the training phase is essential, as it allows the ML model to imitate the expert’s input data, permitting the model to differentiate new inputs based on previously learned training parameters. Logistic Regression (LR) is a simple and efficient method that is easy to understand and interpret. It is also robust and can work well with small datasets using the logistic function, although it may struggle with high-dimensional data or variables that are correlated (Ranganathan et al., 2017). Random Forest (RF) is an ensemble learning method that uses a network of decision trees to handle high-dimensional data and variables that are correlated. It is also robust to overfitting and can work well with missing data. However, RF can be slow and may require high computational resources for large datasets. It can also be difficult to interpret the results and explain the decision-making process because of the majority “vote” approach used for the final decision (Zhu et al., 2018). A decision tree’s (DT) structure is presented as a flowchart, comprising a root, internal nodes, branches, and leaves. The internal nodes are responsible for evaluating the attributes in question, delegating the resulting outcome via branching, and eventually deciding on a final class label for a specific input based on the information gleaned from all attributes. DT can be prone to overfitting and may not generalize well to new data (Hailemariam et al., 2011). Extreme Gradient Boost (XGBoost) and Light Gradient Boost (LGB) are powerful algorithms that can handle large datasets and complex models. They are also efficient at handling missing data, although the results of the model may be hard to explain directly (Ogunleye and Wang, 2019). Gaussian Naive Bayes (GNB) is simple and efficient, with low computational resources required. It can work well with high-dimensional data and may be robust to noise, although it assumes that the data is normally distributed, which may not always be the case. It may also be sensitive to outliers and may struggle with rare events (Malekloo et al., 2022). Support Vector Machine Classifier (SVC) classifies data by defining a hyperplane that maximizes differentiation between two groups by increasing the margin on either side of this hyperplane, employing a kernel function to find nonlinear relationships and enhance the margin. Overfitting is a limitation of the method, and it can also be difficult to tune and interpret the results (Byvatov et al., 2003). K-Nearest Neighbor (KNN) is a simple and efficient algorithm that can handle both classification and regression problems. It can work well with small datasets and can be easily understood and interpreted but it requires a lot of memory and may not work well with high-dimensional data as it is sensitive to the values of k and may be prone to overfitting or underfitting (Song et al., 2017). In addition, we also developed ensemble models using different combinations of the classifiers mentioned above using a soft voting strategy.

Hyperparameters are components of a learning algorithm that must be predefined before model training and fitting. Hyper-parameter tuning/optimization is the process of choosing the set of hyper-parameters that would enhance algorithm performance. Previous studies have used GridSearchCV (grid search cross-validation, cv) for hyper-parameter tuning and model selection, but we found that using the same procedure and dataset for both optimization and evaluation of model performance could lead to data leakage (Hall and Matz, 2020). To address this issue, we used nested cross-validation in our study, applying a distinct routine with two loops: an inner loop (cv = 3) for optimizing model parameters and an outer loop for measuring the optimized model performance on a held-out test set (cv = 5). We believe this approach can reduce the bias compared to GridSearchCV.

After configuring the parameters of each classifier, we evaluated the model performance using various metrics. Specifically, we plotted the Receiver Operating Characteristic (ROC) curve and Calibration plot to assess the discrimination and agreement between the actual observed and predicted values, respectively. In addition, we performed Decision Curve Analysis to examine the clinical usefulness of the model. The ROC curve measures the ability of a classifier to distinguish between positive and negative classes, with the area under the curve (AUC) providing a measure of the classifier’s performance. The calibration plot assesses the agreement between the predicted and observed probabilities across different ranges of predicted probabilities. The Decision Curve Analysis (DCA) evaluates the clinical net benefit of the model by comparing its decision-making performance against alternative strategies.

### 2.6 Model interpretation

The development of machine learning (ML) models for clinical applications should aim to provide interpretable and transparent models to aid in clinical practice. One critical challenge is the so-called “black-box problem,” where models are difficult to interpret, and the reason behind a specific model’s precise prediction for a given patient cohort is unclear. To improve the interpretability of the model, we utilized two methods: SHapley Additive exPlanations (SHAP) and Local Interpretable Model-agnostic Explanations (LIME) (Linardatos et al., 2020; Lundberg et al., 2020). According to SHAP’s computation of feature importance scores, each feature’s average marginal contribution to each prediction—where marginal refers to the difference between the actual predicted value and a base value used as a reference—is calculated. This method was inspired by coalitional game theory (Di Martino and Delmastro, 2022). With a more straightforward interpretable model, LIME approximates a single prediction of a black box model (e.g., decision tree, linear model). However, the simpler model will likely function well locally despite not performing a globally accurate approximation of the complicated model. The prediction is then explained using a simpler model that was learned using the weighted data points (Dindorf et al., 2020).

### 2.7 Web application deployment

To facilitate the utilization of our final selected optimal model, we developed a user-friendly web application using the Python Flask web application development framework and popular frontend techniques (Vogel et al., 2017). The web-based interface allows clinical practitioners and researchers to access and interact with the model straightforwardly and seamlessly. By providing a free and user-friendly interface, we aim to promote the adoption and use of the model in real-world clinical settings. Additionally, the web application includes appropriate security measures to ensure confidential patient data is protected and data privacy is maintained.

### 2.8 Statistical analysis

First, we assessed the normality of the data using the Shapiro-Wilks test. Continuous normal variables were reported as mean values with standard deviation (SD), whereas continuous non-normal variables were reported as median values (interquartile range). Statistical analyses were performed using R Version 4.2.1 (http://www.r-project.org). The ML models were developed and analyzed using Python 3.9.5 and the Scikit-learn package (https://scikit-learn.org). Model performance was assessed using a range of evaluation metrics, including sensitivity, specificity, F1-score, and area under the receiver operating characteristic curve (AUC), which measures the classifier’s ability to distinguish between positive and negative classes. These metrics were selected to provide a thorough assessment of the models’ discrimination and predictive accuracy.

## 3 Results

### 3.1 Patient characteristics

The present study included a total of 540 recipients, with an average age of 59.3 ± 13.5 years 258 people were male and 282 were female. Among the enrolled individuals, prolonged length of stay (PLOS) was defined as P75 of LOS (8 days). The PLOS after surgery were 196 which accounted for 36.3% of the whole cohort. Further details can be found in Table 1.

**TABLE 1:** Baseline of patients’ characteristics.

Characteristics	All (N = 540)	Testing Set (N = 154)	Training Set (N = 386)	*P*
Age (year)	59.3 ± 13.5	59.3 ± 14.2	59.3 ± 13.2	0.970
Gender (n%)				0.187
Female	282 (52.2%)	73 (47.4%)	209 (54.1%)	
Male	258 (47.8%)	81 (52.6%)	177 (45.9%)	
Symptoms Duration	38.5 ± 55.6	35.1 ± 52.2	39.9 ± 57.0	0.341
Affected Limb (n%)				0.358
Both	196 (36.3%)	49 (31.8%)	147 (38.1%)	
Left	173 (32.0%)	51 (33.1%)	122 (31.6%)	
Right	171 (31.7%)	54 (35.1%)	117 (30.3%)	
Muscle Strength (n%)				0.464
3	125 (23.1%)	35 (22.7%)	90 (23.3%)	
4	346 (64.1%)	95 (61.7%)	251 (65.0%)	
5	69 (12.8%)	24 (15.6%)	45 (11.7%)	
Pain (n%)				0.393
Moderate	343 (63.5%)	93 (60.4%)	250 (64.8%)	
Severe	197 (36.5%)	61 (39.6%)	136 (35.2%)	
Hypertension (n%)				1.000
No	295 (54.6%)	84 (54.5%)	211 (54.7%)	
Yes	245 (45.4%)	70 (45.5%)	175 (45.3%)	
DM (n%)				0.143
No	410 (75.9%)	124 (80.5%)	286 (74.1%)	
Yes	130 (24.1%)	30 (19.5%)	100 (25.9%)	
Cardiovascular (n%)				0.423
No	461 (85.4%)	128 (83.1%)	333 (86.3%)	
Yes	79 (14.6%)	26 (16.9%)	53 (13.7%)	
Cerebrovascular (n%)				0.108
No	498 (92.2%)	137 (89.0%)	361 (93.5%)	
Yes	42 (7.78%)	17 (11.0%)	25 (6.48%)	
Hepatic (n%)				0.906
No	480 (88.9%)	136 (88.3%)	344 (89.1%)	
Yes	60 (11.1%)	18 (11.7%)	42 (10.9%)	
Respiratory (n%)				0.406
No	507 (93.9%)	142 (92.2%)	365 (94.6%)	
Yes	33 (6.11%)	12 (7.79%)	21 (5.44%)	
Previous Surgery (n%)				0.692
No	314 (58.1%)	87 (56.5%)	227 (58.8%)	
Yes	226 (41.9%)	67 (43.5%)	159 (41.2%)	
Kidney (n%)				0.694
No	510 (94.4%)	144 (93.5%)	366 (94.8%)	
Yes	30 (5.56%)	10 (6.49%)	20 (5.18%)	
BMI (Kg/m^2^)	25.4 ± 3.70	25.1 ± 3.74	25.5 ± 3.69	0.228
Smoker (n%)				0.774
No	451 (83.5%)	127 (82.5%)	324 (83.9%)	
Yes	89 (16.5%)	27 (17.5%)	62 (16.1%)	
Alcohol (n%)				0.883
No	463 (85.7%)	131 (85.1%)	332 (86.0%)	
Yes	77 (14.3%)	23 (14.9%)	54 (14.0%)	
WBC (10^9/L)	6.63 ± 2.13	6.77 ± 2.61	6.57 ± 1.90	0.372
HB (g/L)	138 ± 16.2	137 ± 16.4	138 ± 16.1	0.910
Platelet (10^9/L)	237 ± 66.7	233 ± 63.1	239 ± 68.0	0.345
ESR (mm/h)	20.3 ± 15.3	20.6 ± 14.7	20.3 ± 15.5	0.832
CRP (mg/L)	6.93 ± 15.2	6.66 ± 13.6	7.03 ± 15.9	0.787
K (mm/h)	3.86 ± 0.35	3.87 ± 0.35	3.86 ± 0.35	0.850
Na (mmol/L)	141 ± 6.03	142 ± 2.30	141 ± 6.99	0.316
Creatinine (μmol/L)	67.4 ± 28.0	65.6 ± 17.1	68.2 ± 31.3	0.219
eGFR (mL/min/1.73m^2^)	94.0 ± 18.0	95.2 ± 18.3	93.5 ± 17.9	0.336
ALB (g/L)	42.2 ± 3.92	41.8 ± 4.18	42.4 ± 3.81	0.159
AST (U/L)	20.6 ± 10.3	20.5 ± 7.75	20.7 ± 11.1	0.818
ALT (U/L	23.8 ± 19.6	24.8 ± 17.4	23.3 ± 20.4	0.417
Day (n%)				0.923
Friday	70 (13.0%)	17 (11.0%)	53 (13.7%)	
Monday	100 (18.5%)	27 (17.5%)	73 (18.9%)	
Saturday	18 (3.33%)	5 (3.25%)	13 (3.37%)	
Sunday	10 (1.85%)	4 (2.60%)	6 (1.55%)	
Thursday	115 (21.3%)	32 (20.8%)	83 (21.5%)	
Tuesday	122 (22.6%)	38 (24.7%)	84 (21.8%)	
Wednesday	105 (19.4%)	31 (20.1%)	74 (19.2%)	
Postoperative drainage 1stDay (mL)	133 ± 163	137 ± 162	131 ± 164	0.685
Operation Duration (n%)				0.529
<150	181 (33.5%)	48 (31.2%)	133 (34.5%)	
≥150	359 (66.5%)	106 (68.8%)	253 (65.5%)	
Infusion volume (mL)	1493 ± 538	1482 ± 530	1498 ± 541	0.759
Blood Loss (mL)				0.702
<200	174 (32.2%)	52 (33.8%)	122 (31.6%)	
≥200	366 (67.8%)	102 (66.2%)	264 (68.4%)	
Segment(s) (n%)				0.479
1	313 (58.0%)	85 (55.2%)	228 (59.1%)	
2	186 (34.4%)	58 (37.7%)	128 (33.2%)	
3	37 (6.85%)	9 (5.84%)	28 (7.25%)	
4	4 (0.74%)	2 (1.30%)	2 (0.52%)	
Transfusion (n%)				0.270
No	484 (89.6%)	134 (87.0%)	350 (90.7%)	
Yes	56 (10.4%)	20 (13.0%)	36 (9.33%)	
LOS (n%)				0.191
Normal	344 (63.7%)	91 (59.1%)	253 (65.5%)	
Prolonged	196 (36.3%)	63 (40.9%)	133 (34.5%)	

BMI, Body mass index (BMI, kg/m^2^); WBC, preoperative white blood cell (WBC, ×10^9/^L); ESR, preoperative erythrocyte sedimentation rate (ESR, mm/h); CRP, preoperative C-reactive protein (CRP, mg/L); Hb, preoperative hemoglobin (Hb, g/L); estimated glomerular filtration rate (eGFR, ml/min/1.73 m^2^) ALB, pre-operative operative albumin (ALB, g/L); AST, preoperative aspartate aminotransferase (AST, U/L); ALT, preoperative alanine aminotransferase (ALT, U/L); LOS, length of stay after surgery.

### 3.2 Variable selection

**FIGURE 2:**
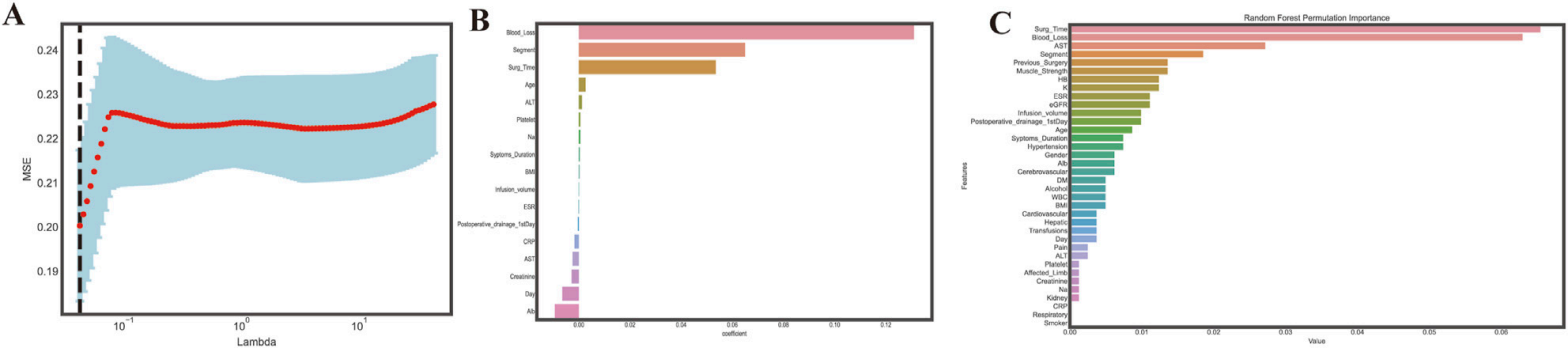
Feature selection. **(A)**. Choosing the best lambda; **(B)**. Features coefficients selected through LASSO; **(C)**. Variables permutations obtained by Random Forest. The random forest permutation importance bar plot assesses the relative significance of input features by calculating the decrease in accuracy caused by randomly rearranging feature values with the most influential feature at the top of the ranking; a bar plotted to represent the impact of each feature shows that the higher the bar corresponding to a feature, the more influential it is in the model.

First, all features were applied to univariate analysis. Then, utilizing the LASSO approach, we eliminated 18 nonzero coefficients from 36 variables. (Figures 2A, B). Meanwhile, we calculated the permutation importance by RF (Figure 2C). Then, after comprehensive consideration of the result of the abovementioned three procedures, we decide to choose: are age, BMI, ESR, ALB, operation duration, infusion volume, blood loss, transfusion, and segments. This feature selection process aimed to reduce the risk of overfitting and improve the model’s generalizability and interpretability.

### 3.3 Model construction

**FIGURE 3:**
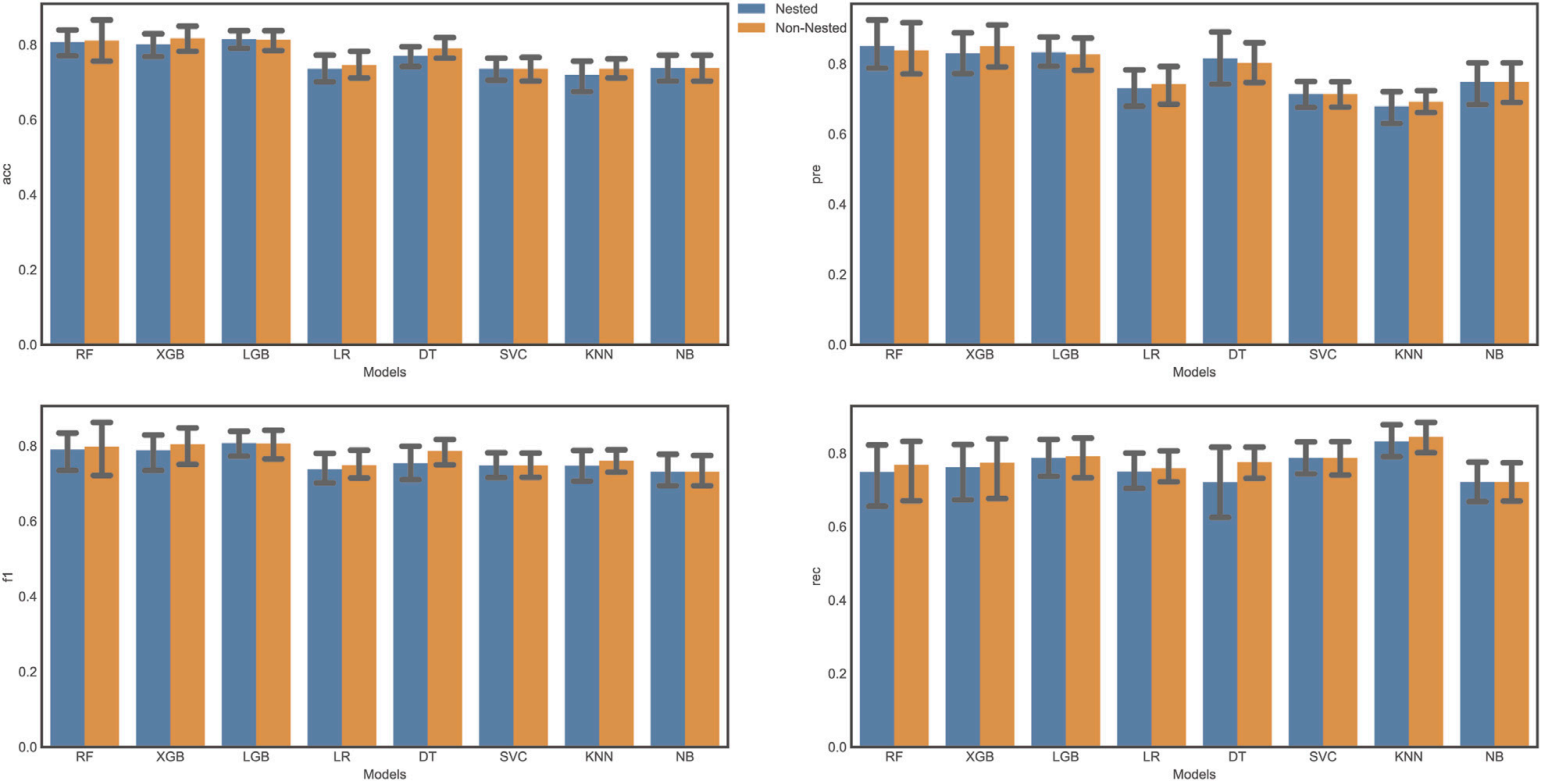
Model Evaluation comparison.

To analyze the bias between the two tuning strategies, we evaluated the performance of classifiers using both grid search with cross-validation and nested cross-validation. We compared several evaluation metrics, including accuracy, precision, F1 score, and recall (Figure 3). Our results indicated that some classifiers showed a higher value for certain metrics (e.g., accuracy) using grid search with cross-validation, but lower values with nested cross-validation. This finding suggests that the grid search approach may result in an overly optimistic score due to data leakage. Consequently, we adopted nested cross-validation for further analysis in this study. Nested cross-validation is a fundamental element of machine learning algorithms, wherein the training data is used to develop a model (f) that maps specific input variables/features (X) to an output/target (Y).

### 3.4 Model performance evaluation

**FIGURE 4:**
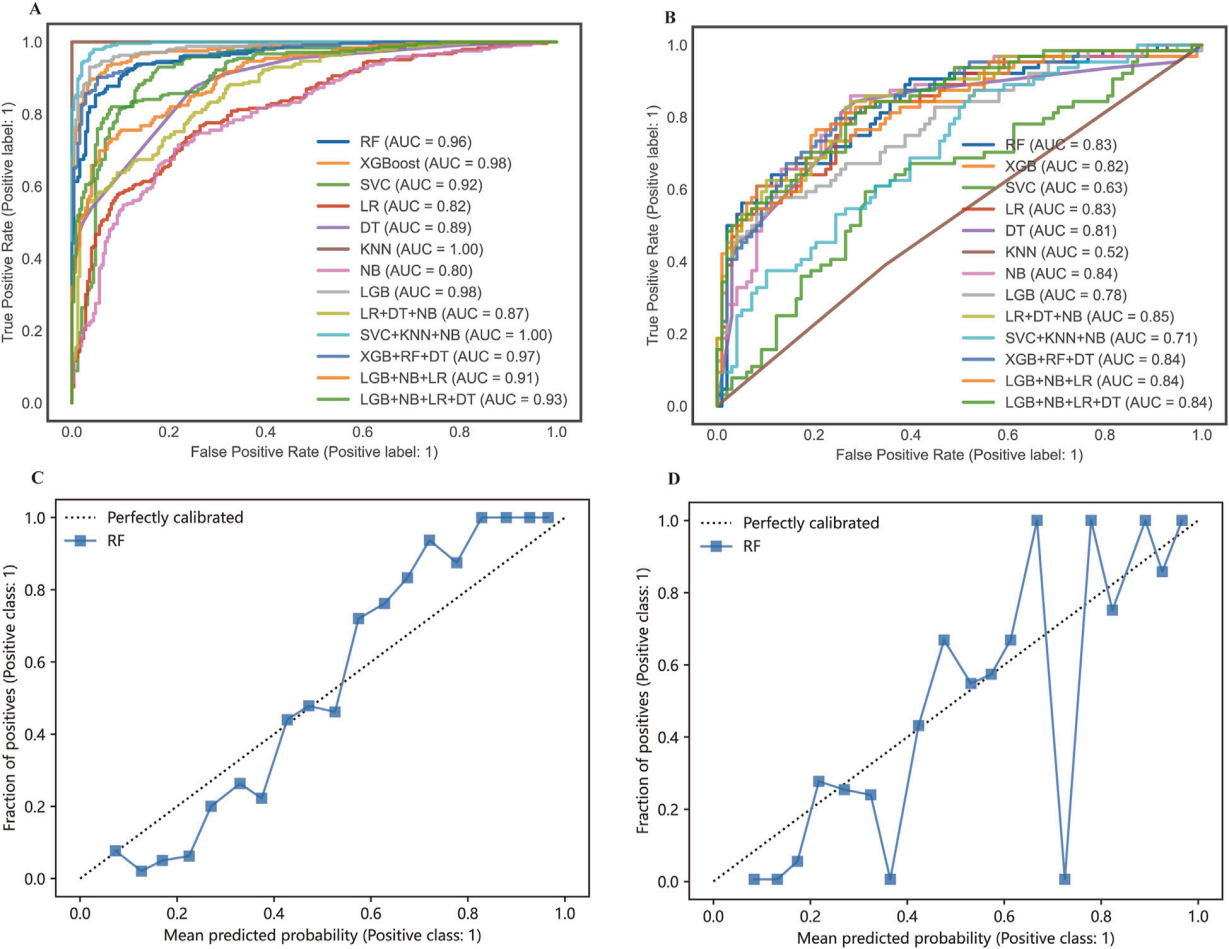
The ROC curves and calibration curves of the training set **(A, C)** and testing set **(B, D)**.

**FIGURE 5:**
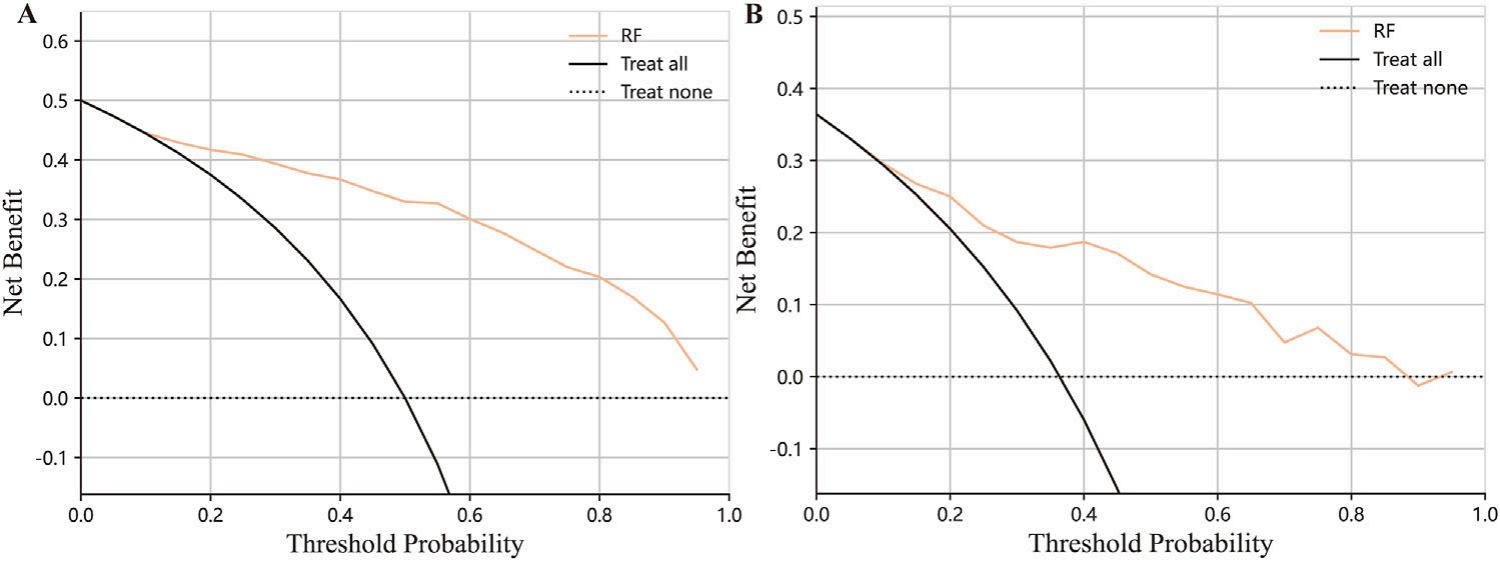
Decision curve analysis on the training set **(A)** and testing set **(B)**.

Receiver operation curves (ROC) of all distinct models were shown in Figures 4A, B, and major evaluation metrics of the testing are displayed in Table 2. LR, RF, DT, XGBoost, LGB, SVC, NB, and KNN achieved AUC in training (testing) set: 0.82 (0.83), 0.96 (0.83), 0.98 (0.82), 0.89 (0.81), 0.98 (0.78), 0.92 (0.63), 0.80 (0.84) and 1.0 (0.52). Besides, the agreement between predicted and observed outcomes was visualized with a calibration plot (Figures 4C, D). The RF model achieved a Brier score of 0.152, a log loss of 0.480, and an accuracy value of 0.784, indicating better agreement between actual and predicted labels while maintaining high accuracy compared to other models or voting strategies. The best hyperparameters combination for the RF included 100 estimators for a balanced number of trees, a maximum depth of six to prevent overfitting, a minimum samples split of five for internal node splitting, the square root of features for classification, and a limit of nine leaf nodes to control complexity (Wang et al., 2021). Therefore, the RF model was chosen for model interpretability analysis. Additionally, we generated a decision curve to display the clinical efficiency of the selected model in practice (Figures 5A, B).

**TABLE 2:** Model performance for predicting PLOS after surgery.

Classifiers	Brier loss	Log loss	Acc	Recall	F1	Sen	Spe	Npv	Ppv
RF	0.152	0.480	0.784	0.531	0.660	0.531	0.949	0.756	0.872
XGB	0.161	0.498	0.772	0.609	0.678	0.609	0.878	0.775	0.765
LGB	0.181	0.545	0.722	0.578	0.622	0.578	0.816	0.748	0.673
LR	0.167	0.500	0.753	0.813	0.722	0.812	0.714	0.854	0.650
SVC	0.243	0.689	0.648	0.563	0.558	0.562	0.704	0.711	0.554
KNN	0.451	15.564	0.549	0.391	0.407	0.391	0.653	0.621	0.424
NB	0.175	0.749	0.759	0.719	0.702	0.719	0.786	0.811	0.687
DT	0.163	1.666	0.747	0.750	0.701	0.750	0.745	0.820	0.658
LR + DT + NB	0.153	0.468	0.747	0.734	0.696	0.734	0.755	0.813	0.662
SVC + KNN + NB	0.214	0.623	0.654	0.531	0.548	0.531	0.735	0.706	0.567
XGB + RF + DT	0.155	0.477	0.747	0.500	0.610	0.500	0.908	0.736	0.780
LGB + NB + LR	0.156	0.476	0.759	0.688	0.693	0.688	0.806	0.798	0.698
LGB + NB + LR + DT	0.152	0.465	0.759	0.609	0.667	0.609	0.857	0.771	0.736

Sen, sensitivity; Spe, specificity; Acc, accuracy; Ppv, positive predictive value; Npv, negative predictive value.

### 3.5 Model interpretation

**FIGURE 6:**
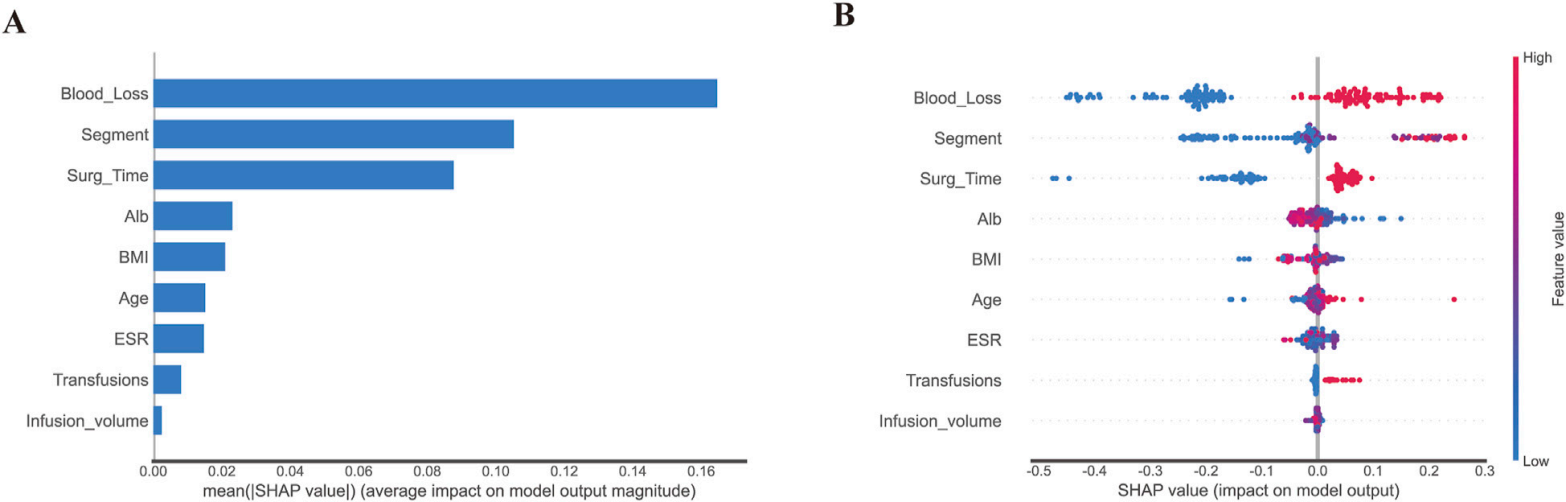
Model interpretation based on Random Forest. **(A)**. The importance of selected variables according to the mean SHAP value; A presentation of variance importance lays out the variables in descending order, with the top variables being the most noteworthy and contributing heavily to the model while displaying greater predictive ability than those placed lower on the list. **(B)**. The selected factors with stability and interpretation. The probability of a patient having a lengthy hospital stay following surgery increases as a feature’s SHAP score rises. A higher value is indicated by the red portion of the feature value. In a summary plot of a final model, the impacts of features on decision-making are depicted alongside feature interactions, where positive SHAP values suggest augmented risks of prolonged length of stay (PLOS) in each prediction, with negative ones pointing towards lowered risks, and higher values reflecting even greater associated risks; colors on the plot represent original feature values, with blue being indicative of lower and red of higher values, and each point corresponding to a patient prediction.

To illustrate the factors that contribute to PLOS in our model, we employed SHAP to identify the most important variables. Figure 6A depicts the top variables ranked by their average absolute SHAP value. The top 20 features of our model are listed in Figure 6B, with the feature rating (i.e., the relevance of the model to the outcome) shown on the y-axis and the SHAP value (i.e., the impact of a specific model component) on the x-axis. The contribution of each feature to the overall prediction is represented by the dots in each feature-important row, with red dots denoting high-risk values and blue dots representing low-risk values. The use of SHAP enables us to better understand the relative importance of different features to the prediction model and how they contribute to the model’s output.

**FIGURE 7:**
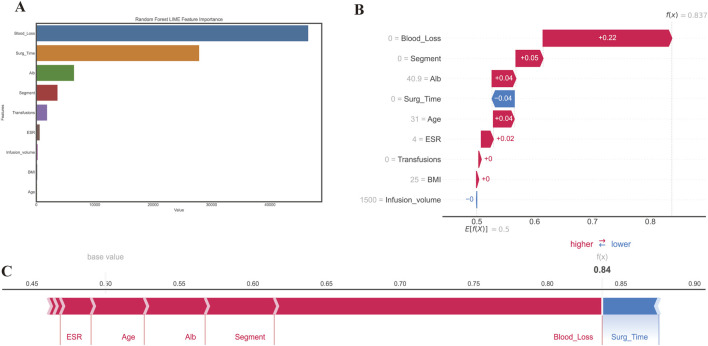
The interpretation using a different method of prediction results from RF. **(A)** LIME method; **(B, C)** SHAP method. An interpretive force plot featuring a specific case from the test set where PLOS was observed, depicts local interpretation, revealing that the model’s predictive capacity was significantly influenced by the blood loss feature, with its effect predominantly contributing towards increasing the model prediction score beyond the base value.

To further enhance the interpretability of our model, we present two standard examples utilizing LIME (Figure 7A) and SHAP (Figures 7B, C) respectively. The LIME and SHAP explainers are both popular tools for interpreting machine learning models, allowing for the identification of input features that have the greatest impact on the model’s output for individual instances. These tools can help to identify the key factors driving model predictions and provide more insight into the decision-making process. For example, we demonstrate a case of a 72-year-old male who underwent spine surgery and experienced a prolonged length of stay (Figures 7A–C).

### 3.6 Web application development

**FIGURE 8:**
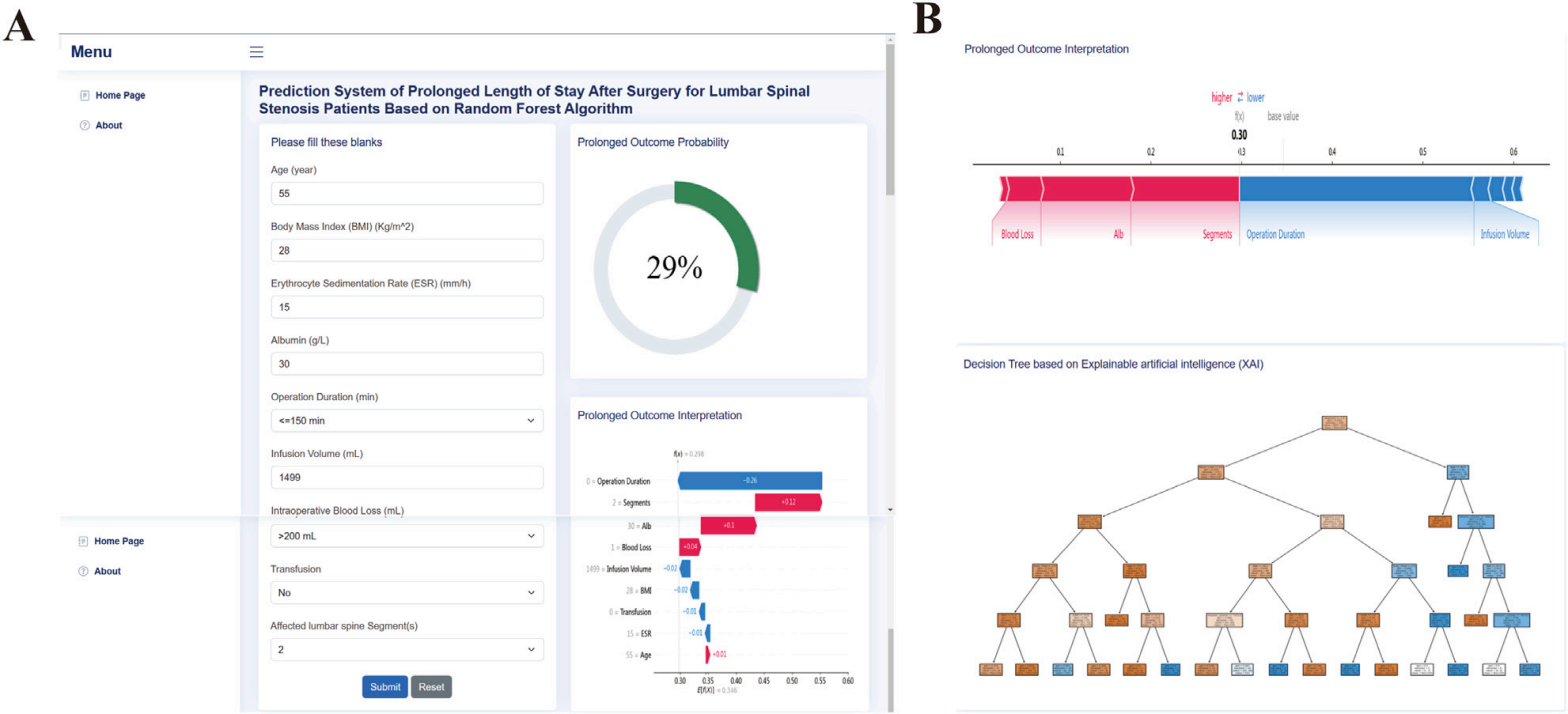
Web application screenshot upper **(A)** and lower **(B)** panel.

We developed a web-based tool to facilitate further research and clinical application of our random forest (RF) model for predicting prolonged length of stay (PLOS) after surgery. The web tool is free and user-friendly and can be accessed at http://43.143.217.126:8090/pplos. The user interface (UI) of the web application is shown in Figure 8 and requires the user to input their age, body mass index (BMI), erythrocyte sedimentation rate (ESR), albumin (ALB) level, operation duration, infusion volume, blood loss, transfusion, and surgical segments. After entering the required information, the user can select the “Predictor” button to obtain a probability estimate of PLOS after surgery and the corresponding interpretation of the result. The web tool can serve as a valuable resource for clinical decision-making and personalized patient care by helping to identify patients at high risk of PLOS and providing guidance for appropriate interventions.

## 4 Discussion

One of the most prevalent orthopedic conditions, lumbar spinal stenosis is also a major health issue and a prevalent ailment in the aging population, with an incidence rate of more than 5% in the aged population globally (Peng et al., 2019). Lower back discomfort, neurogenic nerve impingement, lower limb pain, and impaired walking capacity are among the typical clinical signs. The illness has a substantial influence on both everyday functioning and overall life satisfaction. There is a chance that LSS can cause moderate to severe pain, which can reduce patient quality of life and increase healthcare costs. When it comes to treating individuals with lumbar spinal stenosis, traditional open lumbar decompressive surgery has always been considered the gold standard (Chen et al., 2020; Ulrich et al., 2015). It was reported that adding a fusion procedure based on decompression surgery will increase the risk of the major length of hospitalization complications and resource (Machado et al., 2017). Furthermore, considering the main population of lumbar spinal stenosis are aging people, it is essential to reduce the length of stay (LOS) to reduce the complication risk of pneumonia, thrombolysis, impairment of motor function, etc., where LOS is largely depending on the LOS after surgery.

Recent scholarly research has demonstrated that the adoption of ML techniques has yielded a significant improvement in the performance or predictive accuracy of prognostic models, particularly when compared to conventional statistical or expert-based systems. Specifically, ML methods are a valuable and efficacious tool for classification tasks as they possess the potential to generate prediction models with performances that are comparable to those obtained through conventional statistical methods (Mezher et al., 2022)^,^ (Park et al., 2021). The main difference between machine learning (ML) methods and conventional statistical methods is that ML methods can handle a large number of variables and their interactions simultaneously, whereas conventional statistical methods typically focus on a few variables at a time. ML methods also can learn and improve as they are exposed to more data, whereas conventional statistical methods often require assumptions to be made about the data before analysis. The clinical significance of using ML methods over conventional statistical methods is that ML can identify patterns and relationships in complex and large datasets that may not be easily detected using conventional statistical methods. This can lead to more accurate and personalized diagnoses, treatment plans, and patient outcomes. Additionally, ML methods can be used for prediction and risk stratification, which can enable early intervention and prevention of certain conditions. Overall, ML methods offer the potential to improve clinical decision-making and patient outcomes by utilizing all available data and identifying complex relationships and patterns that may be missed by conventional statistical methods.

Definitions, descriptions, and a framework consisting of four main indispensable steps for the development of ML applications have been provided. Step 1 entails an appraisal of the data’s quality and accessibility; Step 2 necessitates method validation to determine the most effective ML model(s); Upon identification of the optimal ML models, step 3 involves an assessment of their capacity to function collaboratively with other datasets to evaluate generalizability; Finally, step 4 entails real-world evaluations of the data to ascertain performance and generalizability (Rashidi et al., 2019). In brief, upon completion of data collection, cleaning, feature engineering, and selection of an appropriate machine learning approach, the subsequent stage entails the development and validation of models, culminating in the deployment of the final model. However, each step aforementioned above can be divided into several sub-steps. For example, model validation can also be accomplished using multiple methods from simple to complex: “train-test” splitting, general “k-fold” cross-validation, nested cross-validation “leave-one-out” cross-validation, and bootstrapping. In this research, we utilized a nested cross-validation technique.

While several studies have focused on identifying the risk factors and developing models to predict the prolonged length of stay (PLOS) after surgery, few have explored their practical application in clinical practice. With this study, we aim to highlight and visualize the current applicability of machine learning (ML) in predicting PLOS after surgery for lumbar spinal stenosis (LSS) patients. Accurate prediction of PLOS following spine surgery for such patients is crucial for clinical management and optimizing healthcare resource allocation. Our Random Forest (RF) model, identified as the best performer, had an area under the curve (AUC) of 0.83. Furthermore, we offered an extensive interpretation of the decision-making processes and impact of several variables using SHAP values and plots, demonstrating that ML can provide reliable predictions and explanations for LSS patients. We believe that our model, now available as a web application, has the potential to provide useful suggestions and references for orthopedic surgeons managing LSS patients.

Features from electronic healthcare records have been extracted using data mining, and prediction models have been created using machine learning techniques in different subjects (Obermeyer and Emanuel, 2016; Rajkomar et al., 2019; Patel et al., 2018; Hung et al., 2018). Our findings show that the ensemble technique, which employs several single learners to make decisions by voting, is more predictable than a single model such as SVM, KNN, or LR. This suggests that the ensemble technique, as compared to previous models, has greater generalization capabilities for predictions of the occurrence of PLOS following open lumbar spine surgery. Additionally, a mechanism is required that chooses the best machine learning models and improves the ensemble method’s structure using techniques including boosting, bagging, stacking several models, soft voting, etc. (Ribeiro and Coelho, 2020). The ensemble model’s computing and interpretation complexity must also be considered while using a tiny device. Regarding the agreement between the predicted labels and observed labels, in terms of brier loss and log-loss, we selected RF as our final model to predict the occurrence of PLOS after open lumbar spinal surgery.

High dimensional features will add the complexity of model interpretation and may lead to over-fitting in machine learning analysis and it is advantageous to reduce the number of features. In this study, we screened variables via univariate analysis, LASSO, and permutation importance. Feature selection can be divided into three categories, filter method, wrapping method, and embedding method (Bolon-Canedo et al., 2013). In this research, we implemented univariate analysis and LASSO which belong to the filter method and embedding method respectively. Besides, we also considered feature importance. There are two commonly used methods for calculating feature significance scores: permutation importance and *Gini* importance (also known as mean decrease impurity or built-in feature important). The main benefit of this approach is the speed at which the necessary values are computed during the Radom Forest training. The inherent feature significance technique has the propensity to favor (select as significant) numerical features and high-cardinality categorical characteristics. We overcame the shortcomings of the impurity-based feature importance by using permutation importance, which does not favor high-cardinality features and may be computed on a test set that is excluded.

The selected features using the abovementioned methods, are age, BMI, ESR, ALB, operation duration, infusion volume, blood loss, transfusion, and segments. Age and BMI have been demonstrated to be independent risk factors for an extended length of stay in our result, which is in line with previous reports (Yang et al., 2012; Barina et al., 2020; Linder et al., 2022). ESR is a useful laboratory technique for diagnosing inflammatory, neoplastic, and viral disorders. However, it was also found to be a risk factor of PLOS in our study. Kim et al. reported that the length of hospitalization was affected by ESR when regarding infected diabetic foot ulcer patients, which was consistent with our result (Kim et al., 2016). The most prevalent protein in plasma, known as serum albumin (ALB), serves as the primary regulator of both fluid kinds of transport within and across bodily compartments as well as the primary determinant of plasma oncotic pressure. In our result, the lower ALB was associated with longer PLOS. This result has been proved in previous studies (Zhou et al., 2015). Additionally, operation time and intraoperative blood loss were risk factors for a prolonged length of stay connected to surgery (Wu S. et al., 2020). Bian et al. discovered additional risk variables for transfusion, including longer operation time, higher projected intraoperative blood loss, and increased postoperative drainage volume (Bian et al., 2023). This shows that these surgical-related factors are risk factors of each other.

For clinical practitioners to understand the findings and recommendations generated by artificial intelligence (AI) models, interpretability is crucial. One algorithm can never outperform the super learning strategy, which chooses the best regression algorithm from all weighted combinations of a group of candidate algorithms. However, it also complicated the model’s training process and the explanation of the forecast. In this work, SHAP and LIME were used to transform black-box models into understandable visual explanations. This method describes feature contributions at instance levels in addition to feature levels. Orthopedic surgeons will get practical information about how to reduce the avoidable risk of PLOS after surgery at a particular level in practice from the feature-level explanation as well as an understanding of the feature itself. The chances ratio given by conventional logistic regression is unable to produce such knowledge directly. When presented with particular real situations that do not always match the population’s average outcomes, the instance-level explanation will aid physicians in making decisions. An accurate risk management strategy may be possible with such a customized prediction. In our investigation, we also found that our method outperformed conventional statistical analysis in identifying risk factors by uncovering more meaningful characteristics, enabling clinicians to evaluate their plausibility through an explainable approach based on their experience and expertise.

Both SHAP and LIME analyses showed that intraoperative blood loss is the most significant contributor to the patient outcome. To address this, we propose several clinical protocol changes aimed at minimizing blood loss and enhancing patient outcomes. First, adopting minimally invasive surgical techniques can significantly reduce tissue trauma and associated blood loss (Rampersaud et al., 2006). Additionally, the use of advanced hemostatic devices and agents can improve bleeding control during procedures (Hikata et al., 2017). Preoperatively, managing anemia and optimizing fluid protocols can enhance blood volume and maintain hemodynamic stability, further mitigating blood loss (Warner et al., 2020). Intraoperatively, real-time blood loss monitoring technologies can facilitate immediate interventions, such as fluid resuscitation or transfusions, to address excessive bleeding (Rinehart et al., 2012). A multidisciplinary approach, particularly collaboration with anesthesiologists, is crucial for optimizing blood pressure and coagulation status during surgery. Implementing these changes is expected to reduce intraoperative blood loss, potentially leading to shorter recovery times and decreased lengths of stay. Consequently, recalibrating our predictive model will be necessary to reflect these improvements accurately.

Web applications bring convenience for AI spread and daily practicality. Apart from the interpretation of clinical-relevant machine learning models, partibility is meaningful. Thus, we also developed a web application based on the final RF model, where we still focus on the explanation of the result calculated by our model. Noteworthy, to improve the user experience, we deployed AJAX techniques (Marchetto et al., 2008), where web pages are updated asynchronously by exchanging data with a web server behind the scenes. This will allow you to update parts of a web page, without reloading the whole page.

Our web-based machine learning application for PLOS following lumbar spinal stenosis surgery offers valuable clinical benefits. By providing spine surgeons with the ability to assess PLOS risk before surgery, this tool improves decision-making, optimizes surgical planning, and enables more efficient resource allocation. With interpretability features powered by SHAP and LIME, it offers personalized insights into the specific factors influencing a patient’s risk, enabling more precise preoperative counseling and postoperative care strategies. By accurately PLOS in lumbar spinal stenosis surgery, our model enables early identification of high-risk patients. This facilitates targeted interventions, reducing hospital stays and costs while optimizing resource utilization, ultimately enhancing patient outcomes and satisfaction. The insights derived from our model also contribute to continuous quality improvement efforts in surgical departments, allowing surgeons to analyze PLOS trends and outcomes, ultimately identifying opportunities to refine surgical techniques and improve patient education.

## 5 Limitations

This study has several limitations that should be acknowledged. Firstly, Our study was based on electronic medical records from a single medical center, which limits the ability to generalize our findings to broader healthcare settings. Additionally, the absence of external validation using an independent cohort means we cannot fully confirm the robustness and superiority of our model’s performance. To enhance the validity of our conclusions, we recognize the need for larger, prospective trials that evaluate our results across diverse clinical environments and patient populations. Moreover, with the advancement of artificial intelligence, deep learning has shown great potential for improving medical prediction models. Therefore, future research will focus on the development of a deep learning model to predict the risk factors of prolonged length of stay after surgery for patients with lumbar spinal stenosis. Finally, we recognize the potential for overfitting given the single-center design of our study. To address this limitation and reduce any biases in the dataset, our upcoming multi-center research will include a broader and more diverse patient population, enhancing the generalizability and robustness of our findings. We will also aim to incorporate more extensive data and information from various sources to increase the accuracy of our predictions and facilitate clinical decision-making.

## 6 Conclusion

The LSS in patients who underwent open surgery could be accurately assessed and classified in this study using the ML-based risk classification method. Combining ML, SHAP, LIME, and web application may be able to provide an explicit explanation of personalized risk prediction, enabling doctors to comprehend intuitively how important model components affect outcomes. This would assist orthopedic surgeons to make better management decisions.

## Data Availability

The data analyzed in this study is subject to the following licenses/restrictions: Upon reasonable request, the corresponding author of this article will provide unrestricted access to the original data. Requests to access these datasets should be directed to songxinghua19@163.com.

## References

[B1] Barina A. Nardelli M. Gennaro N. Corti M. C. Marchegiani F. Basso C. (2020). Impact of laparoscopic approach on the short-term outcomes of elderly patients with colorectal cancer: a nationwide Italian experience. Surg. Endosc. 34 (10), 4305–4314. 10.1007/s00464-019-07197-9 31617097

[B2] Basques B. A. Varthi A. G. Golinvaux N. S. Bohl D. D. Grauer J. N. (2014). Patient characteristics associated with increased postoperative length of stay and readmission after elective laminectomy for lumbar spinal stenosis. Spine (Phila Pa 1976) 39 (10), 833–840. 10.1097/BRS.0000000000000276 24525996 PMC4006290

[B3] Bian T. Zhang L. Man S. Li H. Dou Y. Zhou Y. (2023). Predisposing factors for allogeneic blood transfusion in patients with ankylosing spondylitis undergoing primary unilateral total hip arthroplasty: a retrospective study. J. Orthop. Surg. Res. 18 (1), 9. 10.1186/s13018-022-03464-z 36597109 PMC9811782

[B4] Bolon-Canedo V. Sanchez-Marono N. Alonso-Betanzos A. (2013). A review of feature selection methods on synthetic data. Knowl. Inf. Syst. 34 (3), 483–519. 10.1007/s10115-012-0487-8

[B5] Byvatov E. Fechner U. Sadowski J. Schneider G. (2003). Comparison of support vector machine and artificial neural network systems for drug/nondrug classification. J. Chem. Inf. Comput. Sci. 43 (6), 1882–1889. 10.1021/ci0341161 14632437

[B6] Cairns K. Deer T. Sayed D. van Noort K. Liang K. (2019). Cost-effectiveness and safety of interspinous process decompression (superion). Pain Med. 20 (Suppl. 2), S2-S8–S8. 10.1093/pm/pnz245 PMC6896024 31808529

[B7] Chen T. Zhou G. Chen Z. Yao X. Liu D. (2020). Biportal endoscopic decompression vs. microscopic decompression for lumbar canal stenosis: a systematic review and meta-analysis. Exp. Ther. Med. 20 (3), 2743–2751. 10.3892/etm.2020.9001 32765769 PMC7401848

[B8] Davis N. Hourigan P. Clarke A. (2017). Transforaminal epidural steroid injection in lumbar spinal stenosis: an observational study with two-year follow-up. Br. J. Neurosurg. 31 (2), 205–208. 10.1080/02688697.2016.1206188 27548310

[B9] Dietz N. Sharma M. Adams S. Alhourani A. Ugiliweneza B. Wang D. (2019). Enhanced recovery after surgery (ERAS) for spine surgery: a systematic review. World Neurosurg. 130, 415–426. 10.1016/j.wneu.2019.06.181 31276851

[B10] Di Martino F. Delmastro F. (2022). Explainable AI for clinical and remote health applications: a survey on tabular and time series data. Artif. Intell. Rev. 56, 5261–5315. 10.1007/s10462-022-10304-3 36320613 PMC9607788

[B11] Dindorf C. Teufl W. Taetz B. Bleser G. Frohlich M. (2020). Interpretability of input representations for gait classification in patients after total hip arthroplasty. Sensors (Basel) 20 (16), 4385. 10.3390/s20164385 32781583 PMC7471970

[B12] Exarchos K. P. Goletsis Y. Fotiadis D. I. (2012). A multiscale and multiparametric approach for modeling the progression of oral cancer. BMC Med. Inf. Decis. Mak. 12, 136. 10.1186/1472-6947-12-136 PMC3560119 23173873

[B13] Fritsch C. G. Ferreira M. L. Maher C. G. Herbert R. D. Pinto R. Z. Koes B. (2017). The clinical course of pain and disability following surgery for spinal stenosis: a systematic review and meta-analysis of cohort studies. Eur. Spine J. 26 (2), 324–335. 10.1007/s00586-016-4668-0 27443531

[B14] Gunzer F. Jantscher M. Hassler E. M. Kau T. Reishofer G. (2022). Reproducibility of artificial intelligence models in computed tomography of the head: a quantitative analysis. Insights Imaging 13 (1), 173. 10.1186/s13244-022-01311-7 36303079 PMC9613832

[B15] Hailemariam E. Goldstein R. Attar R. Khan A. (2011). “Real-time occupancy detection using decision trees with multiple sensor types,” Proceedings of the 2011 symposium on simulation for architecture and urban design.

[B16] Hall A. N. Matz S. C. (2020). Targeting item–level nuances leads to small but robust improvements in personality prediction from digital footprints. Eur. J. Personality 34 (5), 873–884. 10.1002/per.2253

[B17] Hikata T. Isogai N. Shiono Y. Funao H. Okada E. Fujita N. (2017). A retrospective cohort study comparing the safety and efficacy of minimally invasive versus open surgical techniques in the treatment of spinal metastases. Clin. Spine Surg. 30 (8), E1082-E1087–e7. 10.1097/BSD.0000000000000460 27841799

[B18] Hong S. Z. Lynn H. S. (2020). Accuracy of random-forest-based imputation of missing data in the presence of non-normality, non-linearity, and interaction. Bmc Med. Res. Methodol. 20 (1), 199. 10.1186/s12874-020-01080-1 32711455 PMC7382855

[B19] Hung A. J. Chen J. Gill I. S. (2018). Automated performance metrics and machine learning algorithms to measure surgeon performance and anticipate clinical outcomes in robotic surgery. JAMA Surg. 153 (8), 770–771. 10.1001/jamasurg.2018.1512 29926095 PMC9084629

[B20] Jain P. Potdar V. (2021). Frameworks for developing an agro-prosumer community group platform. PeerJ Comput. Sci. 7, e765. 10.7717/peerj-cs.765 PMC8670369 34977347

[B21] Katz J. N. Zimmerman Z. E. Mass H. Makhni M. C. (2022). Diagnosis and management of lumbar spinal stenosis: a review. JAMA 327 (17), 1688–1699. 10.1001/jama.2022.5921 35503342

[B22] Kim T. G. Moon S. Y. Park M. S. Kwon S. S. Jung K. J. Lee T. (2016). Factors affecting length of hospital stay and mortality in infected diabetic foot ulcers undergoing surgical drainage without major amputation. J. Korean Med. Sci. 31 (1), 120–124. 10.3346/jkms.2016.31.1.120 26770047 PMC4712569

[B23] Leidner F. Kurt Yilmaz N. Schiffer C. A. (2019). Target-specific prediction of ligand affinity with structure-based interaction fingerprints. J. Chem. Inf. Model 59 (9), 3679–3691. 10.1021/acs.jcim.9b00457 31381335 PMC6940596

[B24] Linardatos P. Papastefanopoulos V. Kotsiantis S. (2020). Explainable AI: a review of machine learning interpretability methods. Entropy (Basel). 23 (1), 18. 10.3390/e23010018 33375658 PMC7824368

[B25] Linder S. Walle L. Loucas M. Loucas R. Frerichs O. Fansa H. (2022). Enhanced recovery after surgery (ERAS) in DIEP-flap breast reconstructions-A comparison of two reconstructive centers with and without ERAS-protocol. J. Pers. Med. 12 (3), 347. 10.3390/jpm12030347 35330347 PMC8954560

[B26] Lu C. X. Huang Z. B. Chen X. M. Wu X. D. (2022). Predicting prolonged postoperative length of stay risk in patients undergoing lumbar fusion surgery: development and assessment of a novel predictive nomogram. Front. Surg. 9, 925354. 10.3389/fsurg.2022.925354 36051703 PMC9426777

[B27] Lundberg S. M. Erion G. Chen H. DeGrave A. Prutkin J. M. Nair B. (2020). From local explanations to global understanding with explainable AI for trees. Nat. Mach. Intell. 2 (1), 56–67. 10.1038/s42256-019-0138-9 32607472 PMC7326367

[B28] Machado G. C. Maher C. G. Ferreira P. H. Harris I. A. Deyo R. A. McKay D. (2017). Trends, complications, and costs for hospital admission and surgery for lumbar spinal stenosis. Spine (Phila Pa 1976) 42 (22), 1737–1743. 10.1097/BRS.0000000000002207 28441309

[B29] Malekloo A. Ozer E. AlHamaydeh M. Girolami M. (2022). Machine learning and structural health monitoring overview with emerging technology and high-dimensional data source highlights. Struct. Health Monit. 21 (4), 1906–1955. 10.1177/14759217211036880

[B30] Marchetto A. Tonella P. Ricca F. (2008). “State-based testing of Ajax web applications,” 2008 1st international conference on software testing, verification, and validation (IEEE).

[B31] Marko O. Brdar S. Panic M. Sasic I. Despotovic D. Knezevic M. (2017). Portfolio optimization for seed selection in diverse weather scenarios. PLoS One 12 (9), e0184198. 10.1371/journal.pone.0184198 28863173 PMC5580993

[B32] Mezher M. A. Altamimi A. Altamimi R. (2022). An enhanced Genetic Folding algorithm for prostate and breast cancer detection. PeerJ Comput. Sci. 8, e1015. 10.7717/peerj-cs.1015 PMC9299265 35875638

[B33] Nakamura M. Kajiwara Y. Otsuka A. Kimura H. (2013). LVQ-SMOTE - learning vector quantization based synthetic minority over-sampling technique for biomedical data. BioData Min. 6 (1), 16. 10.1186/1756-0381-6-16 24088532 PMC4016036

[B34] Obermeyer Z. Emanuel E. J. (2016). Predicting the future - big data, machine learning, and clinical medicine. N. Engl. J. Med. 375 (13), 1216–1219. 10.1056/NEJMp1606181 27682033 PMC5070532

[B35] Ogunleye A. Wang Q.-G. (2019). XGBoost model for chronic kidney disease diagnosis. IEEE/ACM Trans. Comput. Biol. Bioinforma. 17 (6), 2131–2140. 10.1109/TCBB.2019.2911071 30998478

[B36] Orlenko A. Moore J. H. (2021). A comparison of methods for interpreting random forest models of genetic association in the presence of non-additive interactions. BioData Min. 14 (1), 9. 10.1186/s13040-021-00243-0 33514397 PMC7847145

[B37] Paisley K. Jeffries J. Monroe M. Choma T. (2012). Dispersal pattern of injectate after lumbar interlaminar epidural spinal injection evaluated with computerized tomography. Glob. Spine J. 2 (1), 27–32. 10.1055/s-0032-1307251 PMC3864458 24353943

[B38] Park J. E. Mun S. Lee S. (2021). Metabolic syndrome prediction models using machine learning and sasang constitution type. Evid. Based Complement. Altern. Med. 2021, 8315047. 10.1155/2021/8315047 PMC7886522 33628316

[B39] Patel K. N. Angell T. E. Babiarz J. Barth N. M. Blevins T. Duh Q. Y. (2018). Performance of a genomic sequencing classifier for the preoperative diagnosis of cytologically indeterminate thyroid nodules. JAMA Surg. 153 (9), 817–824. 10.1001/jamasurg.2018.1153 29799911 PMC6583881

[B40] Peng H. Tang G. Zhuang X. Lu S. Bai Y. Xu L. (2019). Minimally invasive spine surgery decreases postoperative pain and inflammation for patients with lumbar spinal stenosis. Exp. Ther. Med. 18 (4), 3032–3036. 10.3892/etm.2019.7917 31555386 PMC6755410

[B41] Phan K. Rao P. J. Ball J. R. Mobbs R. J. (2016). Interspinous process spacers versus traditional decompression for lumbar spinal stenosis: systematic review and meta-analysis. J. Spine Surg. 2 (1), 31–40. 10.21037/jss.2016.01.07 27683693 PMC5039840

[B42] Porche K. Yan S. Mohamed B. Garvan C. Samra R. Melnick K. (2022). Enhanced recovery after surgery (ERAS) improves return of physiological function in frail patients undergoing one-to two-level TLIFs: an observational retrospective cohort study. Spine J. 22 (9), 1513–1522. 10.1016/j.spinee.2022.04.007 35447326 PMC9534035

[B43] Rajkomar A. Dean J. Kohane I. (2019). Machine learning in medicine. N. Engl. J. Med. 380 (14), 1347–1358. 10.1056/NEJMra1814259 30943338

[B44] Rampersaud Y. R. Annand N. Dekutoski M. B. (2006). Use of minimally invasive surgical techniques in the management of thoracolumbar trauma: current concepts. Spine 31 (11 Suppl. l), S96–S102. 10.1097/01.brs.0000218250.51148.5b 16685244

[B45] Ranganathan P. Pramesh C. S. Aggarwal R. (2017). Common pitfalls in statistical analysis: logistic regression. Perspect. Clin. Res. 8 (3), 148–151. 10.4103/picr.PICR_87_17 28828311 PMC5543767

[B46] Rashidi H. H. Tran N. K. Betts E. V. Howell L. P. Green R. (2019). Artificial intelligence and machine learning in pathology: the present landscape of supervised methods. Acad. Pathol. 6, 2374289519873088. 10.1177/2374289519873088 31523704 PMC6727099

[B47] Ravindra V. M. Senglaub S. S. Rattani A. Dewan M. C. Hartl R. Bisson E. (2018). Degenerative lumbar spine disease: estimating global incidence and worldwide volume. Glob. Spine J. 8 (8), 784–794. 10.1177/2192568218770769 PMC6293435 30560029

[B48] Ribeiro M. H. D. Coelho L. D. (2020). Ensemble approach based on bagging, boosting and stacking for short-term prediction in agribusiness time series. Appl. Soft Comput. 86, 105837. 10.1016/j.asoc.2019.105837

[B49] Rinehart J. Liu N. Alexander B. Cannesson M. (2012). Review article: closed-loop systems in anesthesia: is there a potential for closed-loop fluid management and hemodynamic optimization? Anesth. Analg. 114 (1), 130–143. 10.1213/ANE.0b013e318230e9e0 21965362

[B50] Roh M. S. Kucher O. A. Shick K. M. Knolhoff D. R. McGarvey J. S. Peterson S. C. (2020). Intramuscular liposomal bupivacaine decreases length of stay and opioid usage following lumbar spinal fusion. Clin. Spine Surg. 33 (8), E359-E363–E63. 10.1097/BSD.0000000000001006 32427717

[B51] Saravi B. Zink A. Ulkumen S. Couillard-Despres S. Hassel F. Lang G. (2022). Performance of artificial intelligence-based algorithms to predict prolonged length of stay after lumbar decompression surgery. J. Clin. Med. 11 (14), 4050. 10.3390/jcm11144050 35887814 PMC9318293

[B52] Song Y. Liang J. Lu J. Zhao X. (2017). An efficient instance selection algorithm for k nearest neighbor regression. Neurocomputing 251, 26–34. 10.1016/j.neucom.2017.04.018

[B53] Ulrich N. H. Kleinstuck F. Woernle C. M. Antoniadis A. Winklhofer S. Burgstaller J. M. (2015). Clinical outcome in lumbar decompression surgery for spinal canal stenosis in the aged population: a prospective Swiss multicenter cohort study. Spine (Phila Pa 1976) 40 (6), 415–422. 10.1097/BRS.0000000000000765 25774464

[B54] Vasquez M. M. Hu C. Roe D. J. Chen Z. Halonen M. Guerra S. (2016). Least absolute shrinkage and selection operator type methods for the identification of serum biomarkers of overweight and obesity: simulation and application. BMC Med. Res. Methodol. 16 (1), 154. 10.1186/s12874-016-0254-8 27842498 PMC5109787

[B55] Vogel P. Klooster T. Andrikopoulos V. Lungu M. (2017). in A low-effort analytics platform for visualizing evolving flask-based Python web services (IEEE Working Conference on Software Visualization VISSOFT), 18–19.

[B56] Wang K. Tian J. Zheng C. Yang H. Ren J. Liu Y. (2021). Interpretable prediction of 3-year all-cause mortality in patients with heart failure caused by coronary heart disease based on machine learning and SHAP. Comput. Biol. Med. 137, 104813. 10.1016/j.compbiomed.2021.104813 34481185

[B57] Warner M. A. Shore-Lesserson L. Shander A. Patel S. Y. Perelman S. I. Guinn N. R. (2020). Perioperative anemia: prevention, diagnosis, and management throughout the spectrum of perioperative care. Anesth. Analg. 130 (5), 1364–1380. 10.1213/ANE.0000000000004727 32167979

[B58] Won Y. I. Kim C. H. Park H. P. Chung S. G. Yuh W. T. Kwon S. W. (2022). A cost-utility analysis between decompression only and fusion surgery for elderly patients with lumbar spinal stenosis and sagittal imbalance. Sci. Rep. 12 (1), 20408. 10.1038/s41598-022-24784-4 36437360 PMC9701767

[B59] Wu P. H. Kim H. S. Jang I. T. (2020a). Intervertebral disc diseases part 2: a review of the current diagnostic and treatment strategies for intervertebral disc disease. Int. J. Mol. Sci. 21 (6), 2135. 10.3390/ijms21062135 32244936 PMC7139690

[B60] Wu S. Liu J. Liang H. Ma Y. Zhang Y. Liu H. (2020b). Factors influencing the length of stay after mediastinal tumor resection in the setting of an enhanced recovery after surgery (ERAS)-TUBELESS protocol. Ann. Transl. Med. 8 (12), 740. 10.21037/atm-20-287 32647665 PMC7333128

[B61] Yang M. Ahn H. J. Kim J. A. Yu J. M. (2012). Risk score for postoperative complications in thoracic surgery. Korean J. Anesthesiol. 63 (6), 527–532. 10.4097/kjae.2012.63.6.527 23277814 PMC3531532

[B62] Zhang J. Fu X. Lin J. Liu Z. Liu N. Wu B. (2018). Study on damage accumulation and life prediction with loads below fatigue limit based on a modified nonlinear model. Mater. (Basel) 11 (11), 2298. 10.3390/ma11112298 PMC6266591 30453480

[B63] Zhou J. Wang M. Wang H. Chi Q. (2015). Comparison of two nutrition assessment tools in surgical elderly inpatients in Northern China. Nutr. J. 14, 68. 10.1186/s12937-015-0054-8 26170020 PMC4499876

[B64] Zhu M. Xia J. Jin X. Yan M. Cai G. Yan J. (2018). Class weights random forest algorithm for processing class imbalanced medical data. IEEE Access 6, 4641–4652. 10.1109/access.2018.2789428

